# Logic-based Approach and Visualization for the Nuclear Medicine Rescheduling Problem

**DOI:** 10.1007/s10916-025-02301-0

**Published:** 2025-11-22

**Authors:** Cinzia Marte, Marco Mochi, Carmine Dodaro, Giuseppe Galatà, Marco Maratea

**Affiliations:** 1 DeMaCS, University of Calabria, Via P. Bucci, Rende, Cosenza, 87036 Italy; 2 Surgiq srl, Via XII Ottobre 1, Genova, 16100 Italy

**Keywords:** Answer set programming, Logic programming, Digital health

## Abstract

The Nuclear Medicine Scheduling problem consists of assigning patients to a day, on which the patient will undergo the medical check, the preparation, and the actual image detection process. The schedule of the patients should consider their different requirements and the available resources, e.g., varying time required for different diseases and radiopharmaceuticals used, number of injection chairs, and tomographs available. Recently, this problem has been solved using a logic-based approach using the Answer Set Programming (ASP) methodology. However, it may be the case that a computed schedule can not be implemented due to a sudden emergency and/or unavailability of resources, thus rescheduling is needed. In this paper, we present an ASP-based approach to solve such a situation, which we call the Nuclear Medicine Rescheduling problem. Experiments on three scenarios in which rescheduling may be needed, and employing real data from a medium size hospital in Italy, show that our rescheduling solution provides satisfying results even when the concurrent number of emergencies and unavailability is significant. We finally present the design and implementation of a web application for the easy usage of our solutions.

## Introduction

Nuclear Medicine is a medical specialty that uses radiopharmaceuticals, a particular kind of drug containing radioactive elements, to treat or diagnose diseases. According to data by the Italian Ministry of Health, almost 2 millions nuclear medicine exams have been carried on during 2022 in Italy.^1^ The process of treating patients with this technique is complex since it involves multiple resources of the hospital and requires multiple steps of varying time. Moreover, often these drugs contain radioactive elements characterized by short half-lives, meaning that they decay rapidly after their preparation. Thus, the timing should be as precise as possible in order to obtain images of good quality. Addressing this problem effectively is crucial due to the nature of the diagnosed illnesses and treated through nuclear medicine, alongside the significant costs associated with this kind of technique. An efficient, possibly optimal, solution can reduce the waiting time of the patients and can thus increase the effective usage of the resources, avoiding waste of time and resources. Nevertheless, reducing the unnecessary time spent by the patients in the hospital is vital for increasing their satisfaction.

Thus, the Nuclear Medicine Scheduling (NMS) problem consists of assigning patients to a day, on which the patient will undergo the medical check, the preparation, and the actual image detection process. The schedule of the patients considers the different requirements of the patients and the available resources, e.g., varying time required for different procedures and radiopharmaceuticals used, number of injection chairs and tomographs available. We followed the definition of the problem given by Medipass,^2^ a leading provider of technological innovation across cancer care and diagnostic imaging in Italy, in collaboration with SurgiQ,^3^ an Italian company active in planning and scheduling solutions.

Given that complex combinatorial problems, possibly involving optimizations, such as the NMS problem, are usually the target applications of logic-based approaches, and recently in [2] a solution based on Answer Set Programming (ASP), an AI language for knowledge representation and reasoning, has been provided. In the ASP methodology, first the problem is formally represented in terms of logical rules (usually called *encoding*), then the encoding is provided as input, together with the data, to a *solver*, which computes a solution to the original problem. There are several reasons for choosing ASP as our approach. First and foremost, ASP has already been successfully applied to a variety of scheduling problems, including in the healthcare domain (see, e.g., [3–8]). Additionally, ASP offers a simple yet expressive syntax [9], supporting advanced features such as optimization statements and database-inspired constructs like aggregates. It also benefits from an intuitive semantics [10], and is backed by efficient solvers (e.g., [11–13]) capable of addressing complex optimization problems using powerful algorithms [14].

Nevertheless, it may be well the case that a previously viable solution may no longer be feasible for a number of reasons which include sudden emergencies coming to the table and/or the unavailability of resources.

In turn, these situations can produce two different types of rescheduling: If the reason is known prior, the rescheduling is called *offline*, since it does not affect the patients already in the hospital, otherwise it is *online*, since it is created while the patients are in the hospital, but without affecting the patients already under treatment.

In this paper we present an ASP-based approach to solve such problem, that we call Nuclear Medicine Rescheduling (NMR) problem. In particular, we address online rescheduling and analyse three scenarios: (*i*) the reallocation of resources due to emergencies that demand immediate access to equipment or require more time on the resources; (*ii*) the unexpected unavailability of essential resources (e.g., an injection chair or a tomography); and (*iii*) the unavailability of a procedure room. Experiments employing real data provided by Medipass, and related to a medium-size hospital, show that the solution produces satisfying results in terms of both time efficiency and solution quality for all scenarios, even in extreme cases where, e.g., the concurrent number of emergencies and unavailability is up to 20% of the patients involved in the scheduling procedure. We finally present the design and implementation of a web application for the easy usage of our solutions: The application has a front-end in which the user can insert the main parameters of the problem, and can visualize the results, and a back-end, for invoking and running the ASP solver transparently without user’s intervention.

## Problem Description

In this section, we provide an overview of the NMS [2] and the NMR problems [1], in two separate subsections, highlighting their key characteristics and fundamental aspects.

### Nuclear Medicine Scheduling

The NMS problem consists of assigning patients to a day and to a tomograph and/or an injection chair if required by the patient or the specific procedure. In our problem, for each day we consider a set of 120 time slots (TS), each representing 5 minutes. Each patient needs an exam and each exam is linked to a protocol defining the phases and the time required for each phase. We considered 11 different protocols. Each protocol can encompass up to four phases, represented as $(\textrm{p}_1)$ anamnesis, $(\textrm{p}_2)$ medical check, $(\textrm{p}_3)$ radiopharmaceuticals injection and bio-distribution time, and $(\textrm{p}_4)$ image detection. Moreover, each phase can require a different amount of time depending on the exam. Table 1 shows the total time needed by each protocol, the partial time required by each phase, expressed as the number of time slots used, and which protocol requires an infusion chair for the phase $(\textrm{p}_3)$.

**Table 1:** Specifications for each protocol, including the number of time slots (TS) needed for each phase, the total time slots for the entire protocol, and the chair request

Protocol Number	#TS for $\textrm{p}_1$	#TS for $\textrm{p}_2$	#TS for $\textrm{p}_3$	#TS for $\textrm{p}_4$	#TS total	Chair
813	3	2	0	8	13	Not required
814	3	2	0	8	13	Not required
815	2	2	4	6	14	Required
817	2	2	3	7	14	Not required
819	2	2	5	7	16	Required
822	2	2	2	7	13	Not required
823	2	2	10	7	21	Required
824	2	2	5	8	17	Required
827	2	2	2	7	13	Not required
828	3	3	0	7	13	Not required
888	2	2	2	9	15	Required

Due to the high number of phases required by each patient and the variety of the considered protocols, in many clinics, the schedule of the patients is sub-optimal. A sub-optimal schedule is problematic not only because of the high cost of the drugs and machines involved in the exams, but is particularly detrimental for the patients since the order and the time required by each phase, in particular the injection and the bio-distribution time, are fundamental for a proper image detection.

Different clinics have different resource availability and may have different requirements in defining a proper solution. Here we present the criteria followed in the clinic that provided us with the real data of the patients and that we use to define the problem. We considered a clinic with two rooms, each with one tomograph and three injection chairs. We started from a list of patients, each requiring a specific protocol, to be assigned on a day. Moreover, certain protocols impose a daily limit on the number of exams that can be executed on a single tomograph. A proper solution must satisfy the following conditions:

- a starting and an ending time should be assigned to every scheduled patient for each required phase;
- there must be at most two patients concurrently in the medical check phase;
- the injection phase must be done in an injection chair or on a tomograph according to the required protocol;
- each injection chair and tomograph can be used by just one patient at the same time;
- patients requiring an injection chair must be assigned to the tomograph in the same room;
- protocol identified by the id 815 cannot be assigned on the same day and tomograph to more than one patient.

The solution should maximize the number of scheduled patients in the considered days and, to increase the satisfaction of the patients and the effectiveness of the exams, the solution should also try to minimize the unnecessary time spent in the clinic by the patients.

### Nuclear Medicine Rescheduling

The rescheduling problem arises when a previously viable schedule becomes infeasible due to unforeseen disruptions. Given the complexity of the NMS problem, where patients must undergo multiple phases following strict protocols, adapting to changes is essential to ensure accurate imaging results. Common scenarios that may disrupt a previously established schedule are:

(*R*1): the reallocation of resources due to emergencies that demand immediate access to equipment or require more time on the resources;
(*R*2): unexpected unavailability of essential resources (e.g., an injection chair or a tomography);
(*R*3): unavailability of a procedure room.

In general, these scenarios require rescheduling the patients to ensure the continuity of medical processes, safeguarding both the quality of patient care and the accuracy of diagnostic outcomes. Moreover, to ensure that all the patients are treated on the originally scheduled day, it is possible to allow overtime usage. Overtime can be implemented by extending working hours beyond the standard schedule, for example by having medical staff stay longer or by adjusting shift rotations to accommodate the increased workload. To address these challenges, a responsive rescheduling solution is essential. Such a solution should be capable of quickly adjusting to changes by reallocating resources. The resulting rescheduling process should prioritize the following desiderata, in descending order:

- assigning as soon as possible the new registrations having an emergency;
- minimizing the difference between the old and the new starting time of the treatment for each rescheduled patient;
- minimizing the time slots assigned in overtime;
- minimizing the difference between the old and the new assigned resources to the patients.

## Formal Problem Definition

In this section, we review the mathematical formulation of the NMS [2], and present the one for the NMR problem, defining the key sets and variables used in our model, in two separate subsections.

### NMS Mathematical Formulation

Let *N* be the set of reservation numbers, *D* be the set of days, and $\textit{TS}=\{1, \dots , 120\}$ denote the set of time slots. Let *R* denote the set of rooms and $S = T \cup C \cup \{\varepsilon \}$ be the set representing the available resources, given by the union of the set *T* of tomographs, the set *C* of chairs and the element $\varepsilon$ denoting that a resource is not required. Let $\textit{PR}$ be the set collecting the protocol numbers referring to exams and $\widetilde{PR}$ a subset of it containing only the protocols with a limit on the number of exams that can be executed on a tomograph for that protocol. Each protocol may comprise a maximum of four phases, as introduced in Section “Nuclear Medicine Scheduling”, represented by the set $P = \{\textrm{p}_1,\textrm{p}_2,\textrm{p}_3,\textrm{p}_4\}$. Moreover, let:

- $\lambda : T \times { \widetilde{PR} } \rightarrow \mathbb {N}$ be the function that returns the maximum number of exams that can be executed on a tomograph of a specific protocol, for all the protocols that require a limitation;
- $\omega : P \times \textit{PR}\rightarrow \mathbb {N}$ be the function that returns the number of time slots required for each phase of a specific protocol;
- $\beta : \textit{PR}\rightarrow \{0,1\}$ be the function that assigns value 1 if the protocol requires both a chair and a tomograph, 0 otherwise;
- $\alpha : S \times R \rightarrow \{0,1\}$ be the function that assigns value 1 if there is an association between the resource and the room, 0 otherwise. Furthermore, let $\textsf{A} = \alpha ^{-1}(1)$ be the set collecting all the good associations between resource and room.

To improve the readability, we specify that we denote elements of $\textit{PR}$ and $\textit{TS}$ with *x* and *y*, respectively. To associate a reservation number, a day, and a protocol, we now introduce the notion of *registration*.

#### Definition 1

A registration $\rho$ is a function of the form $\rho : N \times D \times \textit{PR}\rightarrow \{0,1\}$ such that $\rho (n,d,x) = 1$ if there exists a reservation *n* on a day *d* for the protocol *x*, 0 otherwise. Let $\textsf{R} = \rho ^{-1}(1) = \{ (n,d,x) \in N \times D \times \textit{PR}\mid \rho (n,d,x)=1 \}$ be the set collecting all the registrations.

Consequently, we define the notion of *assignment* to link together a registration with a specific phase of the protocol under consideration and a time slot.

#### Definition 2

An assignment $$\tau$$ is a function of the form $$\tau : \textsf{R} \times P \times \textit{TS}\rightarrow \{0,1\}$$ such that $$\tau ((n,d,x),p,y)=1$$ if a phase *p* and an initial time slot *y* are assigned to a registration, 0 otherwise. Let $$\textsf{T} = \tau ^{-1}(1) = \{((n,d,x),p,y)$$$$\in R \times P \times \textit{TS}\mid \tau ((n,d,x),p,y)=1\}$$ be the set collecting all the assignments.

Before presenting the main problem of this paper, we define the concept of *scheduling*, which connects an assignment with a resource and its allocation.

#### Definition 3

A scheduling $$\sigma$$ is a function of the form $$\sigma : \textsf{T} \times \textsf{A} \rightarrow \{0,1\}$$ such that $$\sigma ((n,d,x,p,y),(s,r))=1$$ if a resource *s* and a room *r* are linked to an assignment, 0 otherwise. The set $$\textsf{S} = \sigma ^{-1}(1) = \{((n,d,x,p,y),(s,r))$$$$\in \textsf{T} \times \textsf{A} \mid \sigma ((n,d,x,p,y),(s,r))=1 \}$$ collects all the tuples eligible as scheduling.

We can now define the *Nuclear Medicine Scheduling* problem.

#### Definition 4

The Nuclear Medicine Scheduling (NMS) problem is defined as the problem of finding a set $\psi$ of tuples $(n,d,x,p,y,s,r) \in \textsf{S}$ that satisfies the following conditions:

($$c_1$$): $$\forall d \in D, \ \forall y \in \textit{TS}, \ \forall (s,r)$$$$\in \textsf{A} \ |\{(n,d,x,p,y,s,r) \in \psi : p\ne p_1\}| \le 1$$;
($$c_2$$): $$\forall d \in D, \ \forall y \in \textit{TS}, \ \forall (s,r)$$$$\in \textsf{A} \ |\{(n,d,x,p,y,s,r) \in \psi : p=p_1\}| \le 2$$;
($c_3$): $\forall x \in \textit{PR}: \beta (x)=1$, it holds that $(n,d,x,p',y',s',r')$ and $(n,d,x,p'',y'',s'',r'')$, with $y' \ne y''$, $s' \in C$, and $s'' \in T$, belong to $\psi$ iff $r' = r''$;
($$c_4$$): $$\forall d \in D, \ \forall x \in \widetilde{PR} , \forall (s,r)$$$$\in \textsf{A} \ |\{(n,d,x,p,y,s,r)$$$$\in \psi : s=t \}| \le \lambda (t,x)$$;
($c_5$): $(n,d,x,\textrm{p}_i,y',s',r)$ and $(n,d,x,\textrm{p}_{i+1},y'',s'',r)$ belong to $\psi$ iff $y'' \ge y' + \omega (\textrm{p}_i,x)$
($c_6$): $\forall (n,d,x,p,y,s,r) \in \psi$ it holds that $y + \omega (p,x) \in TS$
($$c_7$$): $$\forall (n,d,x,p,y,s,r) \in \psi$$

- if $p=\textrm{p}_1$ it holds that $s = \varepsilon$,
- if $p \in \{\textrm{p}_2, \textrm{p}_3\}$ and $\beta (x) = 1$ it holds that $s \in C$,
- if $p \in \{\textrm{p}_2, \textrm{p}_3\}$ and $\beta (x) = 0$ it holds that $s \in T$,
- if $p=\textrm{p}_4$ it holds that $s \in T$.

The specified conditions are necessary to enforce the following constraints: $(c_1)$ each resource (chair or tomograph) can be used by at most one patient at a time; $(c_2)$ at most two patients at a time are allowed during the anamnesis phase; $(c_3)$ patients requiring an injection chair must be assigned to the tomograph of the same room; $(c_4)$ the number of protocols executed on a single tomograph is limited; $(c_5)$ given two consecutive phases $\textrm{p}_i$ and $\textrm{p}_{i+1}$ for a patient, the initial time slot of $\textrm{p}_{i+1}$ must be consistent with respect to $\textrm{p}_i$, i.e., $\textrm{p}_{i+1}$ must start after that $\textrm{p}_i$ has terminated; $(c_6)$ each schedule must not exceed the available time slot; $(c_7)$ the resources must be well distributed, i.e., the phase anamnesis does not include any resource, phases 2 and 3 may require a chair or a tomograph depending on the protocol, and the last phase requires the usage of a tomograph.

Finally, we focus on the notion of optimal solution, which relies on the idle time spent by a patient. Given $(n, d, x, \textrm{p}_1, y', s', r)$ and $(n, d, x, \textrm{p}_4, y'', s'', r) \in \psi$, let $\textit{Htime}(n) = (y'' + \omega (\textrm{p}_4,x)) - y')$ be the time spent by the patient in the hospital deriving from the scheduling and let $\textit{Rtime}(n) = \sum _{p \in P} \omega (p,x)$ be the minimum time required to execute the protocol. Accordingly, we provide the definition of *dominating solution*.

#### Definition 5

Let $\delta _{\psi }$ be the sum over all $n \in N$ of $(\textit{Htime}(n) - \textit{Rtime}(n))$, representing the sum of the differences between the time allocated by the solution for that protocol and the actual time required by a protocol, for each registration number *n*. Additionally, let $\Sigma _{\psi } = \{ (n, d, x, p, y, s, r) \in \psi \}$ represent the set of all elements in a solution $\psi$. A solution $\psi$ dominates a solution $\psi '$ if

- $$|\Sigma _{\psi '}| < |\Sigma _{\psi }|$$, or if
- $$|\Sigma _{\psi '}| = |\Sigma _{\psi }| \Rightarrow \delta _{\psi } < \delta _{\psi '}$$.

Now we can define the notion of *maximal scheduling solution*.

#### Definition 6

A scheduling solution is maximal if any other scheduling solution does not dominate it.

### NMR Mathematical Formulation

Following the description presented in Section “Nuclear Medicine Rescheduling”, we introduce the set *E* of emergencies and the set $O=\{121, \dots , 150\}$ of time slots in overtime to model the scenarios that may lead to a rescheduling need. Moreover, consider the functions:

- $\eta : E \times D \times \textit{PR} \times P \rightarrow \textit{TS}$, which assigns a specific time slot to an emergency occurring in a day for a specific phase of some protocol;
- $\nu _1: S \times D \rightarrow \{0,1\}$, which returns 1 if the resource is out of service on that day;
- $\nu _2: R \times D \rightarrow \{0,1\}$, which returns 1 if the room is not accessible on that day.

Consequently, we introduce the extended set of reservation numbers, including emergencies, $\widetilde{N} = N \cup E$, and the extended set of time slots, incorporating overtime, $\widetilde{\textit{TS}} = \textit{TS} \cup O$. We then define the registration and assignment functions, $\widetilde{\rho }$ and $\widetilde{\tau }$ respectively, which operate analogously to those introduced in Definitions 1 and 2. The only difference is that they operate over the modified sets $\widetilde{N}$ and $\widetilde{\textit{TS}}$, instead of the original sets *N* and $\textit{TS}$. Taking these elements into account, we proceed to define the concept of *rescheduling*.

#### Definition 7

A rescheduling $$\widetilde{\sigma }$$ is a function of the form $$\widetilde{\sigma }: \widetilde{\textsf{T}} \times \textsf{A} \rightarrow \{0,1\}$$ such that $$\widetilde{\sigma }((n,d,x,p,y),(s,r))=1$$ if there is an assignment paired with a resource and a room such that $$\nu _1(s,d) = 0$$ and $$\nu _2(r,d)=0$$, 0 otherwise. The set $$\widetilde{\textsf{S}} = \widetilde{\sigma }^{-1}(1) = \{((n,d,x,p,y),(s,r))$$$$\in \widetilde{\textsf{T}} \times \textsf{A} \mid \widetilde{\sigma }((n,d,x,p,y),(s,r))=1 \}$$ collects all the tuples suitable for the rescheduling.

Building upon the definition of rescheduling, we now define the *Nuclear Medicine Rescheduling* problem.

#### Definition 8

The Nuclear Medicine Rescheduling (NMR) problem is defined as the problem of finding a set $\widetilde{\psi }$ of tuples $(n,d,x,p,y,s,r) \in \widetilde{\textsf{S}}$ that satisfies all the conditions of Definition 4 according to the extended sets.

Finally, we analyze the new optimization criteria detailed in Section “Problem Description”. Since these criteria depend on specific components of the tuple that defines the rescheduling, we use the projection function $\pi _i$ to directly access the desired element. Specifically, $\pi _i$ takes an element $\textbf{t} = (n,d,x,p,y,s,r) \in \widetilde{\textsf{S}}$ and returns the $i^\textit{th}$ element of the tuple, e.g., $\pi _3(\textbf{t})=x$.

Now, consider a solution $\psi$ of the NMS problem and a solution $\widetilde{\psi }$ of the NMR problem, then we define:

(*i*): $$o^4_{\widetilde{\psi }}:= \displaystyle \sum _{\tilde{\textbf{t}} \in \widetilde{\psi } \, \ n \in E}$$$$|\pi _5(\tilde{\textbf{t}}) - \eta (n,d,x,p)|$$;
(*ii*): $$o^3_{\psi , \widetilde{\psi }}:= \displaystyle \sum _{\tilde{\textbf{t}} \in \widetilde{\psi } \, \ n \in N}$$$$|\pi _5(\tilde{\textbf{t}}) - \pi _5(\textbf{t})|$$;
(*iii*): $$o^2_{\widetilde{\psi }}:= |\tilde{\textbf{t}} \in \widetilde{\psi } \, \ \pi _5(\tilde{\textbf{t}})$$$$\in O \vee \pi _5(\tilde{\textbf{t}}) + \omega (p,x) \in O|$$;
(*iv*): $$o^1_{\psi , \widetilde{\psi }}:= |\tilde{\textbf{t}} \in \widetilde{\psi } \, \ \exists \ \textbf{t}$$$$\in \psi \ \textit{s.t.} \ \pi _1(\textbf{t})=\pi _1(\tilde{\textbf{t}}),$$$$\pi _3(\textbf{t})=\pi _3(\tilde{\textbf{t}}), \pi _4(\textbf{t})=$$$$\pi _4(\tilde{\textbf{t}}), \pi _6(\textbf{t})=\pi _6(\tilde{\textbf{t}})|$$.

With (*i*) we compute the overall difference between the required and assigned time slots across all emergencies; with (*ii*) we calculate the overall difference between the original and rescheduled starting time slots for all patients whose appointments have been modified from the initial schedule; with (*iii*) we count how many tuples in the rescheduling refer to time slots in overtime; with (*iv*) we count the resources that have not been assigned differently from the initial schedule.

With these elements in place, we are ready to define the notion of dominating solution for the NMR problem.

#### Definition 9

Let $\psi$ represent a solution of the NMS problem and $\tilde{\psi }'$ and $\tilde{\psi }''$ denote two solutions of the NMR problem. We say that $\tilde{\psi }''$ is dominated by $\tilde{\psi }'$ if:

- $$o^4_{\widetilde{\psi }'} < o^4_{\widetilde{\psi }''}$$, or if
- $$o^4_{\widetilde{\psi }'} = o^4_{\widetilde{\psi }''} \Rightarrow o^3_{\psi , \widetilde{\psi }'} < o^3_{\psi , \widetilde{\psi }''}$$, or if
- $o^4_{\widetilde{\psi }'} = o^4_{\widetilde{\psi }''}$ and $o^3_{\psi , \widetilde{\psi }'} = o^3_{\psi , \widetilde{\psi }''} \Rightarrow o^2_{\widetilde{\psi }'} < o^2_{\widetilde{\psi }''}$, or if
- $$o^4_{\widetilde{\psi }'} = o^4_{\widetilde{\psi }''}$$ and $$o^3_{\psi , \widetilde{\psi }'} = o^3_{\psi , \widetilde{\psi }''}$$ and $$o^2_{\widetilde{\psi }'} = o^2_{\widetilde{\psi }''} \Rightarrow o^1_{\psi , \widetilde{\psi }'}> o^1_{\psi , \widetilde{\psi }''}$$.

#### Definition 10

A solution $\psi$ is optimal if it is not dominated by any other rescheduling solution.

## Background on ASP

Answer Set Programming (ASP) [10] is a programming paradigm developed in the field of non-monotonic reasoning and logic programming. In this section, we first overview the language of ASP, by presenting its syntax and semantics in two separate subsections. Then, the last subsection introduces the ASP programming methodology. More detailed descriptions and a more formal account of ASP, including the features of the language employed in this paper, can be found in [9, 10]. Hereafter, we assume the reader is familiar with logic programming conventions.

### Syntax

The syntax of ASP is similar to that of Prolog. Variables are strings starting with an uppercase letter, and constants are non-negative integers or strings starting with lowercase letters. A *term* is either a variable or a constant. A *standard atom* is an expression $p(t_1, \ldots , t_n)$, where *p* is a *predicate* of arity *n* and $t_1, \ldots , t_n$ are terms. An atom $p(t_1, \ldots , t_n)$ is ground if $t_1, \ldots , t_n$ are constants. A *ground set* is a set of pairs of the form $\langle consts\!:\!conj \rangle$, where *consts* is a list of constants and *conj* is a conjunction of ground standard atoms. A *symbolic set* is a set specified syntactically as $\{Terms_1: Conj_1; \cdots ; Terms_t: Conj_t \}$, where $t>0$, and for all $i \in [1,t]$, each $Terms_i$ is a list of terms such that $|Terms_i| = k> 0$, and each $Conj_i$ is a conjunction of standard atoms. A *set term* is either a symbolic set or a ground set. Intuitively, a set term $\{X\!:\! a(X,c), p(X);Y\!:\! b(Y,m)\}$ stands for the union of two sets: The first one contains the *X*-values making the conjunction *a*(*X*, *c*), *p*(*X*) true, and the second one contains the *Y*-values making the conjunction *b*(*Y*, *m*) true. An *aggregate function* is of the form *f*(*S*), where *S* is a set term, and *f* is an *aggregate function symbol*. Basically, aggregate functions map multisets of constants to a constant, e.g., the function *#count* computes the number of terms, while function *#min* returns the minimum of integers.

An *aggregate atom* is of the form $f(S) \prec T$, where *f*(*S*) is an aggregate function, $\prec \ \in \{<, \le ,>, \ge , \ne , =\}$ is a operator, and *T* is a term called guard. An aggregate atom $f(S) \prec T$ is ground if *T* is a constant and *S* is a ground set. An *atom* is either a standard atom or an aggregate atom. A *rule* *r* has the following form:

$$\begin{aligned} a_1 \ | \ \ldots \ | \ a_n \ :\!- \ b_1,\ldots , b_k, not\ b_{k+1},\ldots , not\ b_m. \end{aligned}$$

where $a_1,\ldots ,a_n$ are standard atoms, $b_1,\ldots ,b_k$ are atoms, $b_{k+1},\ldots ,b_m$ are standard atoms, and $n,k,m\ge 0$. A literal is either a standard atom *a* or its negation $not\ \ a$. The disjunction $a_1 \ | \ldots \ | \ a_n$ is the *head* of *r*, while the conjunction $b_1, \ldots , b_k, not\ b_{k+1},$ $\ldots , not\ b_m$ is its *body*. Rules with empty body are called *facts*. Rules with empty head are called *constraints*. A variable that appears uniquely in set terms of a rule *r* is said to be *local* in *r*, otherwise it is a *global* variable of *r*. An ASP program is a set of *safe* rules, where a rule *r* is *safe* if the following conditions hold: *(i)* for each global variable *X* of *r* there is a positive standard atom $\ell$ in the body of *r* such that *X* appears in $\ell$, and *(ii)* each local variable of *r* appearing in a symbolic set $\{ \textit{Terms}\!:\! \textit{Conj}\}$ also appears in a positive atom in $\textit{Conj}$.

A *weak constraint* [15] $\omega$ is of the form:

$$\begin{aligned} :\sim b_1,\ldots , b_k, not\ b_{k+1},\ldots , not\ b_m.\ [w@l] \end{aligned}$$

where *w* and *l* are the weight and level of $\omega$, respectively. (Intuitively, [*w*@*l*] is read as “weight *w* at level *l*”, where the weight is the “cost” of violating the condition in the body of *w*, whereas levels can be specified for defining a priority among preference criteria). An ASP program with weak constraints is $\Pi = \langle P,W \rangle$, where *P* is a program and *W* is a set of weak constraints.

A standard atom, a literal, a rule, a program or a weak constraint is *ground* if no variables appear in it.

#### Syntactic Shortcuts

In the following, we also use *choice rules* of the form $\{p\}$, where *p* is an atom. Choice rules can be viewed as a syntactic shortcut for the rule $p\ | \ p'$, where $p'$ is a fresh new atom not appearing elsewhere in the program, meaning that the atom *p* can be chosen as true.

#### Example 1

In this example, we report a rule with an aggregate, a choice rule, a constraint, and a weak constraint, respectively, that will be part of the ASP encoding for the NMR problem.

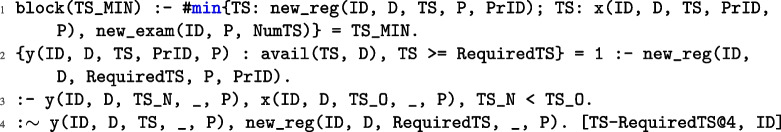



### Semantics

Let *P* be an ASP program. The *Herbrand universe* $U_{P}$ and the *Herbrand base* $B _{P}$ of *P* are defined as usual. The ground instantiation $G_P$ of *P* is the set of all the ground instances of rules of *P* that can be obtained by substituting variables with constants from $U_{P}$.

An *interpretation* *I* for *P* is a subset *I* of $B_{P}$. A ground literal $\ell$ (resp., $not\ \ell$) is true w.r.t. *I* if $\ell \in I$ (resp., $\ell \not \in I$), and false (resp., true) otherwise. An aggregate atom is true w.r.t. *I* if the evaluation of its aggregate function (i.e., the result of the application of *f* on the multiset *S*) with respect to *I* satisfies the guard; otherwise, it is false.

A ground rule *r* is *satisfied* by *I* if at least one atom in the head is true w.r.t. *I* whenever all conjuncts of the body of *r* are true w.r.t. *I*.

A model is an interpretation that satisfies all rules of a program. Given a ground program $G_P$ and an interpretation *I*, the *reduct* [16] of $G_P$ w.r.t. *I* is the subset $G_P^I$ of $G_P$ obtained by deleting from $G_P$ the rules in which a body literal is false w.r.t. *I*. An interpretation *I* for *P* is an *answer set* (or stable model) for *P* if *I* is a minimal model (under subset inclusion) of $G_P^I$ (i.e., *I* is a minimal model for $G_P^I$) [16].

Given a program with weak constraints $\Pi = \langle P,W \rangle$, the semantics of $\Pi$ extends from the basic case defined above. Thus, let $G_{\Pi } = \langle G_P,G_W \rangle$ be the instantiation of $\Pi$; a constraint $\omega \in G_W$ is violated by an interpretation *I* if all the literals in $\omega$ are true w.r.t. *I*. An *optimum answer set* for $\Pi$ is an answer set of $G_P$ that minimizes the sum of the weights of the violated weak constraints in $G_W$ in a prioritized way.

#### Example 2

In the following we explain the semantics of the rules introduced in Example 1 (we refer to the rule at line *i* with $r_i$): ($r_1$) is a rule with aggregate #min that derives the atom *block* that gets the first time slot where a new registration or the delay of a registration appears; ($r_2$) is a choice rule that assigns a starting time for the first phase to all the new registrations, ensuring that the assigned starting time is after the required time slot; ($r_3$) is a constraint that ensures that all the starting times assigned in the reschedule are not before the previously assigned ones; and ($r_{4}$) minimizes, at level 4, the delay in assigning new registrations that have an emergency. The semantics of each rule will become more clear in the context of the whole encoding presented in the next section.

### Programming Methodology

**Fig. 1:**
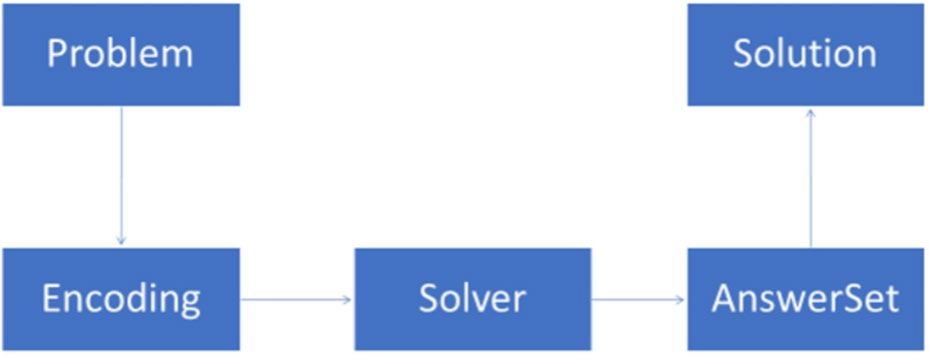
ASP programming methodology schema

Figure 1 depicts a representation of a solution based on a logic-based declarative programming approach, as our ASP solution, consisting of five blocks as described in the following:

- Problem: this block represents the problem description or formulation to be modeled and solved.
- Encoding: this block involves the formal representation of the problem, using ASP in our case, based on the informal description of the problem or the precise mathematical formulation provided.
- Solver: this block takes the encoding of the problem as input and generates the solutions, in our case answer sets.
- AnswerSet: this block represents the output of the solver and corresponds to the set of atoms that satisfy all the rules of the encoding, according to the semantics given above.
- Solution: this block is the solution of the problem, in which the answer sets are interpreted as solutions of the input problem.

The presence of a clear programming methodology is, arguably, one of the advantages that ASP offers. Other advantages, in comparison to alternative logic-based formalisms, include: (*i*) The ASP high-level specifications are declarative and often appreciated even by non-experts since they find them readable, differently from the specifications employed by the other formalisms, e.g., Propositional Satisfiability (SAT) and Constraint Programming (CP). (*ii*) There are free and open source systems (like clingo [11], or wasp [13]), whose performances are often comparable to those of industrial tools for Integer Linear Programming, like, e.g., cplex, or to gurobi, or SAT solvers. (*iii*) ASP allows for easily expressing and reasoning on multi-objective and multi-level optimizations, which is not the case for, e.g., optimization variants of SAT such as Max-SAT (unless weights having exponential gaps are applied).

## ASP Encoding for the NMR problem

In this section, we present our ASP solution designed to model the NMR problem, using the input language of clingo [11]. We refer the reader to Section 5 of the work by [2] for the encoding of the NMS problem. The presentation is organized in two subsections, in which the former deals with the representation of the input data, while the latter contains the ASP encoding.

### Data Model

The input data is specified by means of the following atoms:

- instances of avail(TS,D) denote that the time slots TS is available on day D;
- instances of exam(PrID,P,NumTS) denote the features of an exam, where PrID denotes the exam protocol number, P indicates the phase, and NumTS specifies the time required for that phase in terms of the number of time slots;
- instances of tomograph(T,R) and chair(C,R) denote the allocation of the tomograph T and the chair C to the room R, respectively;
- instances of on(PrID,T) denote that protocol PrID must be executed on tomograph T;
- instances of required_chair(PrID) denote the necessity of a chair for the phases 1 and 2 for the protocol PrID;
- instances of cost(PrID, NumTS) denote the total duration in terms of time slots, denoted as NumTS, of the phases within the protocol identified by PrID;
- instances of limit(PrID,N) denote the maximum number N of exams with protocol number PrID that can be executed on a fixed tomograph in a day.
- instances of new_reg(ID,RequiredTS,P,PrID) denote new registrations to be assigned, where ID is the identification number, which should be scheduled as close as possible to the time slot RequiredTS, P is the required phase, and PrID is the protocol number to be followed;
- instances of new_exam(ID,P,NumTS) denote the delay of a registration with identification number ID, where phase P requires additional NumTS time slots to complete the treatment;
- instances of unavailable_chair(ID,DAY) and unavailable_tomograph(ID,DAY) denote that the chair (resp., tomograph) with identifier ID is not available on day DAY;
- instances of unavailable_room(ROOM, DAY, TS) denote a room ROOM not available on day DAY in the time slot TS;
- instances of x(ID,D,TS,PrID,P) denote part of the solution of the NMS problem, where the registration with identification number ID for the exam with protocol number PrID, regarding the phase P, has been scheduled for the day D during the time slot TS;
- instances of chair(C,ID,D,TS) and tomograph(T,ID,D,TS) denote part of the solution of the NMS problem, denoting the resource (either the chair C or the tomograph T, respectively) allocated to the patient ID on the day D for the time slot TS.

The output is the rescheduling, consisting of atoms similar to x, tomograph, and chair detailed above, but denoted as y, y_tomograph, and y_chair, respectively.

### Encoding of the NMR Problem

**Fig. 2:**
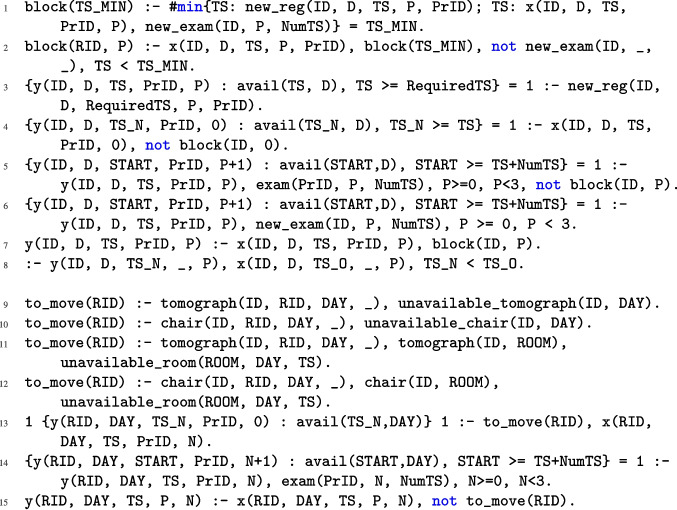
ASP encoding of NMR problem

**Fig. 3:**
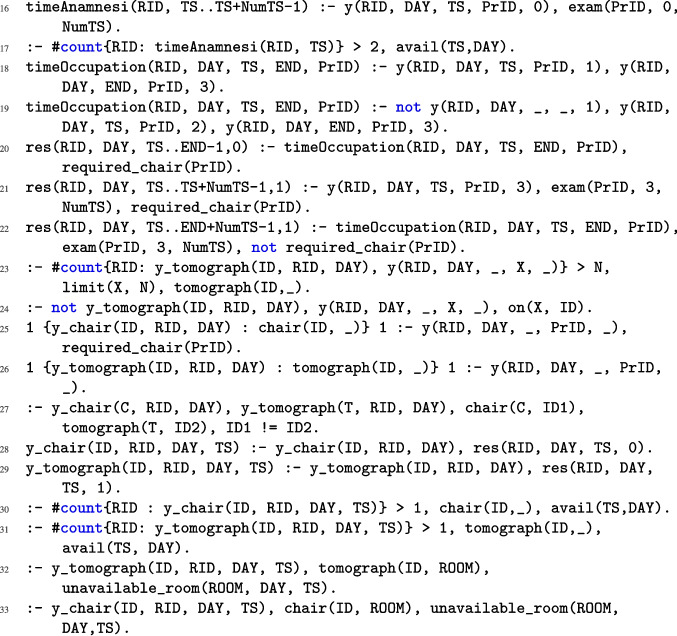
ASP encoding of NMR problem (continued)

**Fig. 4:**
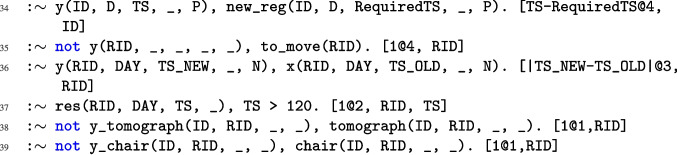
ASP encoding of NMR problem (continued)

As outlined in Section “Problem Description”, rescheduling may result from various scenarios. Below, we present the complete ASP encoding used to model the rescheduling process. To simplify the description, we denote by $r_i$ the rule appearing at line *i* of Figs. 2, 3, and 4. Specifically, the code block from lines $r_1$ to $r_8$ encodes scenario *R*1, which involves the reallocation of resources due to emergencies that require immediate access to equipment or require more time on the resources. The block from lines $r_9$ to $r_{15}$ corresponds to scenarios *R*2 (characterized by rules $r_9, r_{10}, r_{13}, r_{14}$, and $r_{15}$) and *R*3 (characterized by rules $r_{11}$ to $r_{15}$), which address the unexpected unavailability of essential resources (e.g., an injection chair or a tomograph) and the unavailability of a procedure room, respectively. Then, the block from rules $r_{16}$ to $r_{33}$ encodes the assignment of resources and introduces some auxiliary atoms used to encode all the constraints characterizing the NMR problem, as formalized in Definition 8. Finally, the rules from $r_{34}$ to $r_{39}$ are weak constraints that are used to reach the optimal solution and are ordered in a prioritized way, accordingly to Definition 9. A detailed explanation of each rule follows.

Rule $r_1$ derives an auxiliary atom that identifies the minimum (i.e., earliest) time slot in which either a new registration is scheduled or a delay of an existing registration occurs. Then, in rule $r_2$, another auxiliary atom is derived, getting all the registrations and phases starting before the derived time slot. Rule $r_3$ assigns a starting time for the first phase to all the new registrations, ensuring that the assigned starting time is after the required time slot. Rules $r_4$ and $r_5$ assign the first and the subsequent phases to the previously assigned patients that are not involved in delays and were assigned after the first delay or arrival of a new registration. Rule $r_6$ assigns a starting time for all the phases of the registrations involved in the delays, ensuring that the subsequent phases are assigned following the new required time. Rule $r_7$ assigns the same starting time as in the original schedule to the registrations and phases previously derived by the auxiliary atom. Rule $r_8$ ensures that all the starting times assigned in the reschedule are not before the previously assigned ones.

Rules $r_9$ and $r_{10}$ state that a patient RID assigned to a tomograph (resp., chair) ID on a given DAY has to be moved if that tomograph (resp., a chair) is unavailable on that day. Rules $r_{11}$ and $r_{12}$ state that a patient RID assigned to a tomograph (resp., a chair) located in a room ROOM that is unavailable has to be moved. Rule $r_{13}$ assigns a new starting time slot to each patient who was originally scheduled but needs to be moved. Rule $r_{14}$ keeps an already scheduled session for a given phase *N* and assigns to its subsequent planned phases a starting time slot, under the condition that the start of the phase does not extend beyond the latest available time slot for a session on that day. Rule $r_{15}$ includes in the rescheduling all originally scheduled patients that must not be moved.

Rule $r_{16}$ keeps track of the time slots allocated to a patient during phase 0 via the auxiliary atom timeAnamnesis(ID,TS). Rule $r_{17}$ restricts the number of patients during the anamnesis phase to a maximum of two. Rules $r_{18}$ and $r_{19}$ generate the auxiliary atom timeOccupation(ID, D, TS, END, PrID), for protocols that require phase 1 and those that do not, respectively. This atom represents the duration needed for each patient ID from the initial time slot TS of phase 1 (phase 2, resp.) to the first one END of phase 3, concerning the protocol PrID on the day D. Rule $r_{20}$ produces the auxiliary atom res(ID,D,TS,0) for each time slot derived from the previous rule. Specifically, the constant 0 denotes that a chair is required for each of these time slots. Rules $r_{21}$ and $r_{22}$ produce the atom res(ID,D,TS,1), which differs from the previous one for the constant 1, indicating the use of a tomograph. Specifically, from rule $r_{21}$, it is inferred that a tomograph is employed during phase 3, whereas rule $r_{22}$ indicates the tomograph’s usage in phases 1 and 2, according to the atom timeOccupation. Rule $r_{23}$ ensures that the limit of protocols that can be executed on a single tomograph is respected. Rule $r_{24}$ ensures that it is not possible for a patient to be assigned to a tomograph different from the one assigned to the specific protocol. Rule $r_{25}$ produces the atom y_chair(C,ID,D) representing the assignment of a chair C on the day D to the patient ID when the protocol PrID requires a chair. Rule $r_{26}$ produces the atom y_tomograph(T,ID,D) similar to the previous but considering the tomography, representing the assignment of a tomograph T on the day D to the patient ID. Rule $r_{27}$ prevents the patient who moves from the chair to the tomograph from changing rooms. Rule $r_{28}$ and $r_{29}$ generate the atoms y_chair(C,ID,D,TS) and y_tomograph(T,ID,D,TS), respectively, indicating the time slots TS during which the chair C and tomograph T are used by the patients ID on the day D. Rules $r_{30}$ and $r_{31}$ ensure that at most one patient is assigned to each tomograph and chair in every time slot, respectively. Rules $r_{32}$ and $r_{33}$ ensure that no tomograph (or chair, respectively) is assigned if it is located in an unavailable room.

Rule $r_{34}$ minimizes the delay in assigning new registrations that have an emergency. Rule $r_{35}$ minimizes the number of patients that have to be moved and are not assigned to the rescheduled. Rule $r_{36}$ minimizes the difference between the old and the new starting time of the treatment for each rescheduled patient. Rule $r_{37}$ minimizes the time slots assigned in overtime. Rules $r_{38}$ and $r_{39}$ minimize the difference between the old and new resources assigned to the patients.

## NMR Experimental Results

In this section, we report the results of an empirical analysis of the NMR problem via ASP. We performed experiments on an Apple M1 CPU @ 3.22 GHz machine with 8 GB of physical RAM. The ASP system used was clingo [11] 5.6.2, with a timeout of 5 minutes, and the parameters*--opt--strategy=usc* for faster optimization and*--parallel-mode 4* for parallel execution: This setting is the results of a preliminary analysis with various options. The ASP encoding and the instances employed in this section can be found at: https://github.com/CinziaMarte/JoMS2025.

### NMR Benchmarks

We used real data provided by Medipass, collected from a medium-sized hospital, to compute solutions to the NMS problem. In more detail, we tested instances covering over a year of daily examinations. We tested 366 instances, each corresponding to weekdays, resulting in a total of 72 weeks. The solution schedules the patients for a day, divided into a 10-hour window split into 120 time slots of 5 minutes each. Every patient is linked to one of the possible exams. In particular, protocol “823” is required by more than 85% of the patients, thus, the majority of the patients need an exam protocol that requires 2 time slots for the anamnesis and other 2 time slots for the medical preparation, 10 time slots for the drug injection and the bio-distribution time and, at last, the image detection requires 7 time slots. The other patients can be associated with one of the other 10 possible protocols. The schedule is done considering two rooms for the radiotherapy, each equipped with a tomograph and three chairs. The number of patients requiring an exam varies daily, with an average of 29 patients per day and a maximum of 37. The scheduling solutions subsequently served as input for solving the NMR problem. We randomly selected three representative schedules, each corresponding to a distinct case labeled as *low*, *medium*, and *high*. These cases reflect different levels of treatment reallocation: in the *low* case, treatments were assigned to a small number of registrations (about 30); in the *medium* case, to a moderate number (about 80); and in the *high* case, to a large number (about 120). This variation directly impacts the flexibility of the rescheduling process, as a lower number of registrations leaves more time slots available for rearrangement, while a higher number restricts rescheduling options. For scenario *R*1, we generated a total of 9 instances: 3 with an increasing number of new registrations with an emergency, 3 with an increasing number of patients with delays, and 3 combining both types of events. This resulted in 27 instances overall across the three scenarios. To simulate the factors that require rescheduling, we proceed as follows: for a new registration with an emergency, we randomly select a protocol among the possible ones, a preferred time slot, and the starting phase of the registration. For patients with delays, we randomly select a scheduled patient and a required phase and assign a new treatment duration needed to complete the phase. Similarly, we simulate the unavailability of either a resource or a room. In scenario *R*2, we randomly select a resource (either a chair or a tomograph) that was assigned in the initial schedule and mark it as unavailable. The number of unavailable resources is gradually increasing from one to three. In scenario *R*3, we randomly select a room and then define three progressively larger time slot ranges labeled as *small*, *moderate*, and *large*, during which the room becomes unavailable. The number of time slots in each range is randomly selected. Specifically, for the case with a low number of registrations in input, the unavailability spans 3, 5, or 7 time slots; for the medium case, it spans 5, 10, or 15 time slots; for the large case 5 time slots.

**Table 2:** Scenario *R*1: Analysis of the time required to optimally compute rescheduling considering an increasing number of new registrations (NR) (rows) and registrations with delay (D) (columns)

	0 D	1 D	2 D	3 D
0 NR		0.1/0.4/4.7 s	1.1/3.8/4.2 s	0.5/8.2/8.5 s
1 NR	0.2/0.3/2.9 s	1.4/1.3/6.5 s		
2 NR	0.6/3.4/3.6 s		0.3/2.7/3.5 s	
3 NR	0.5/3.4/3.7 s			0.4/4.5/12.5 s

Each cell displays the time required, measured in seconds, for a case with a low/medium/high number of registrations in input

### Results

We present the results obtained from testing our solution to the NMR problem, obtained through the usage of the ASP encoding presented in Section “ASP Encoding for the NMR problem” and using the instances generated as previously discussed. Our analysis comprises an examination of the time required to compute the solution and an evaluation of its quality.

#### Time Efficiency

We present an analysis of the performance of our solution in terms of the time required to optimally solve all considered instances. Specifically, the analysis focuses on the impact of unforeseen events in scenarios *R*1, *R*2, and *R*3, as reported in Tables 2, 3, and 4, respectively. Each scenario is evaluated under varying input sizes, categorized as low, medium, and high. If no solution exists for a given instance, it is marked as *unsat* in the corresponding table.

Table 2 reports results for scenario *R*1. In this table, each row corresponds to a specific number of new registrations, while the columns represent the number of patients experiencing delays. As shown in the table, our solution is able to optimally solve all the instances within the timeout. This result is of particular importance because, in an online scheduling scenario, the operator requesting the reschedule can not afford to wait for a long time before the final result. A closer examination of the instances reveals that those involving only one type of issue (either emergencies or delays) can be solved in less than 10 seconds. When mixing the different kinds of issues, and considering the low and medium scenarios, all the instances still reach an optimal solution in less than 5 seconds. Only in the scenario with a high number of registrations and an almost full schedule could the rescheduling process take more than 10 seconds to find the optimal solution. Notably, this longer runtime occurs just in the instance with 3 new registrations and 3 patients with delays. This instance is particularly complex since it means that approximately 20% of the patients are either new or have encountered an issue.

Table 3 reports results for scenario *R*2, where a variable number of resources (either chairs or tomographs) become unavailable. The results are reported for three different input sizes: low, medium, and high, corresponding to increasing numbers of patient registrations. Each row represents a different level of resource unavailability, ranging from one to three unavailable units. The table shows that for low and medium input sizes, the rescheduling process remains computationally efficient across all configurations, with execution times remaining under 7 seconds. For high input size, results are available only for the case with a single unavailable resource, which required 10.8 seconds to compute.

Table 4 reports results for scenario *R*3, in which a progressively increasing number of time slots is made unavailable for a selected room. The analysis considers three different input sizes: low, medium, and high, corresponding to an increase in the number of registrations. The level of unavailability is classified as low, medium, or high, based on the number of affected time slots. As shown, the rescheduling can compute a solution efficiently for low and medium input sizes when room unavailability is limited. However, for the high input size, the problem instance is unsatisfiable due to the limited number of rooms (only two are available), which becomes insufficient to accommodate all patients when a significant portion of a room’s time is blocked. This behavior is expected, as increasing demand and resource constraints naturally reduce the feasibility of the scheduling problem.

**Table 3:** Scenario *R*2: Analysis of the time required, measured in seconds, to optimally compute rescheduling considering an increasing number of unavailable resources for a case with a low, medium, and high number of registrations in input

Unavailable resources	Low	Medium	High
1	2 s	3.5 s	10.8 s
2	2.5 s	4.1 s	*unsat*
3	2.6 s	6.1 s	*unsat*

**Table 4:** Scenario *R*3: Analysis of the time required, measured in seconds, to optimally compute rescheduling considering an increasing number of unavailable time slots for a specific room for a case with a low, medium, and high number of registrations in input

Unavailable TS per room	Low	Medium	High
Small	2.6 s	4.5 s	*unsat*
Moderate	3 s	190 s	*unsat*
Large	3 s	74 s	*unsat*

#### Solutions Quality

To assess the quality of the rescheduling, we focus on different evaluation criteria depending on the scenario. For scenario *R*1, we consider two key indicators: the number of time slots a new registration must wait before starting treatment (*emergency wait*), and the deviation in start time between the original schedule and the rescheduled one (*change*). For scenarios *R*2 and *R*3, the evaluation is based solely on the *change* indicator, as no new registrations are introduced in these settings.

Table 5 presents the results for all instances involving at least one new registration. The table highlights the total difference in time slots between the required and assigned starting time slots for new registrations, as well as the difference between the scheduling and rescheduling for patients without delays. In a simpler initial case, where a low number of patients are already scheduled, the results show that it is possible to schedule the new registrations as requested without unnecessarily modifying the original schedule. Notably, similar results can be achieved even when the original schedule has a higher number of patients. Indeed, in the medium case, all the solutions assign the new registrations at the required time slot and, only in the most complex instance, the obtained reschedule differs from the original schedule by 12 time slots.

In the high case, finding a solution that perfectly aligns with the original schedule becomes more challenging. Specifically, for new registrations, the rescheduled solution fails to assign the required time slot in 2 out of the 6 tested instances. In these instances, the new registrations have to wait, in total, 5 time slots, thus, with an average waiting time per patient of 12.5 and 8.3 minutes, respectively. However, in instances where optimal solutions include patients with delays, new registrations are assigned to the required time slots. This derives from the randomness of the inputs. In these instances, even if there are more new registrations, the rescheduler can assign them to the required time slots since these new random patients are required to be assigned to a later phase, reducing the total required occupations of the resources. Even in the high case, the approach is still able to reach an optimal solution that is very similar to the original schedule. Indeed, in the instances without any delays, the new solution differs by at most 2 time slots from the original one. Only in the most complex instance, comprising 3 new registrations and 3 patients with delays, the new solution differs by more than 10 time slots. This is due to the high number of registrations already assigned in the original schedule and the fact that all the new registrations are assigned to the required time slot, meaning that the registrations previously assigned in that time slots are forced to be moved. Finally, it is interesting to analyze the usage of overtime. Overtime refers to scheduling beyond the regular time slots to fit in additional or rescheduled patients, which can affect efficiency and resource allocation. Across all cases and instances, the majority of the instances are solved without using overtime, demonstrating the effectiveness of the rescheduling approach. However, in the most complex cases, there may be a limited use of overtime to achieve an optimal solution. Even in these cases, the amount of overtime needed never exceeds 6 time slots. This indicates that the rescheduling process remains efficient, reducing both changes to the original schedule and the need for overtime.

Tables 6 and 7 present the results of varying levels of resource and time slot unavailability, measured in terms of the difference between the original and rescheduled start times. For scenarios involving unavailable resources, no schedule deviations are observed for low and medium input sizes, even as the number of unavailable resources increases from one to three. However, in the case of high input size, 15 patients experienced a shift in their scheduled time. In the case of unavailable time slots, the number of affected patients increases proportionally with the level of unavailability.

**Table 5:** Scenario *R*1: Analysis of the sum of time slots required to promptly assign the new registrations with emergency (*Emerg. Wait*) and the difference between scheduling and rescheduling (*Change*) for patients without delay in each instance

Instance	Low		Medium		High	
	Emerg. Wait	Change	Emerg. Wait	Change	Emerg. Wait	Change
1 NR	0	0	0	0	0	0
2 NR	0	0	0	0	5	0
3 NR	0	0	0	0	5	2
1 NR + 1D	0	0	0	0	0	0
2 NR + 2D	0	0	0	0	0	7
3 NR + 3D	0	0	0	12	0	16

The instances are characterized by the number of new registrations (NR) and registrations with delay (D), and are evaluated across three different cases with a low, medium, and high number of registrations in input

**Table 6:** Scenario *R*2: Analysis in terms of differences between the scheduling and rescheduling of the patients

Unavailable resources	Low	Medium	High
1	0	0	15
2	0	0	*unsat*
3	0	0	*unsat*

The instances are defined by varying levels of unavailability of resources and are evaluated across three different cases with a low, medium, and high number of registrations in input

**Table 7:** Scenario *R*3: Analysis in terms of differences between the scheduling and rescheduling of the patients

Unavailable TS per room	Low	Medium	High
Small	1	3	*unsat*
Moderate	2	5	*unsat*
Large	3	6	*unsat*

The instances are defined by varying the number of unavailable time slots (categorized as small, moderate, and large) and are evaluated across three different cases with a low, medium, and high number of registrations in input

## Web Application

**Fig. 5:**
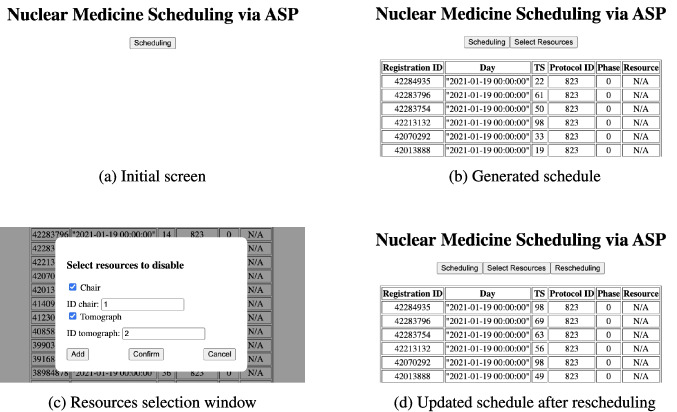
Web application for Nuclear Medicine Rescheduling via ASP

The ASP solution presented in this work is accessible through a dedicated web interface, designed to facilitate interaction with the underlying ASP encoding. This interface supports dynamic user engagement and enables the handling of unforeseen events, such as the temporary unavailability of resources, that necessitate rescheduling operations. We have integrated the ASP encoding and the clingo solver within a Node.js framework, while also developing a graphical user interface (GUI) for it. This approach enables the solver to be incorporated into a user-friendly web application, eliminating the challenges that non-expert users may face when installing and managing the solver. In our prototype, users can already freely configure the parameters that represent the unforeseen situation. Figure 5 illustrates the web interface, which includes both initial scheduling and the subsequent rescheduling. In particular, we exemplify its usage on scenario *R*2, in which an unforeseen event makes either a chair or a tomograph unavailable. More specifically, Figure 5a illustrates the initial state of the web application, where users are presented with a minimal interface featuring a single action button labeled *Scheduling*. This starts the generation of a feasible schedule based on predefined constraints and input data. Figure 5b presents the computed schedule in tabular format, shown after the scheduling process has been completed. Each row represents a scheduled patient and includes attributes such as patient ID, date, time slot, protocol, phase, and assigned resource. At this stage, the interface offers an additional option *Select Resources* to enable further interaction. Figure 5c presents the dialog through which users can specify unavailable resources. This includes entering resource identifiers and dynamically confirming selections, enabling a scenario for rescheduling. Finally, Fig. 5d shows the updated schedule following the execution of the *Rescheduling* function. The system recomputes a valid scheduling that incorporates the modified constraints, thus demonstrating its capacity for adaptive planning in response to unforeseen conditions.

## Related Work

This paper is an extended and revised version of [1], having the following main additions: (*i*) a high-level, mathematical formulation of the NMR problem (Section “NMR Mathematical Formulation”, which is independent from the executable encoding employed, in our case expressed with ASP), (*ii*) the ASP encoding (Section “Encoding of the NMR Problem”) and a related experimental analysis (Section “Results”) of scenarios *R*2 and *R*3, and (*iii*) a web application system for easy usage of our solution (Section “Web Application”).

In the rest of this section, we first analyse papers that consider approaches to the problem we are facing, then we mention works in which ASP has been employed for solving rescheduling problems.

In [17] the authors report their experience with rescheduling nonurgent imaging and procedures during the pandemic. To this aim, the authors conducted daily virtual huddles with discussions of rescheduling strategies and issue tracking, reviewing all cases using radiologists, schedulers, residents, and administrative leadership. In [18] the authors conducted a cross-sectional study on data obtained via magnetic resonance imaging schedule reviews and self-administrated questionnaires to estimate the rate of “No-Shows” or “Reschedule” magnetic resonance imaging appointments and investigate the correlating factors. Even if in these studies emerged the need for a rescheduling solution to overcome emergencies and issues in the planned schedule, and in some works such as [19–23] the scheduling problem is studied and solved, from our understanding no works proposed and evaluated a solution to the rescheduling problem, as we are doing in the current paper.

As we mentioned in the introduction, ASP has been successfully used for solving hard combinatorial and application scheduling problems in several research areas, including the Healthcare domain (see, e.g., [24] for a survey). Focusing on the problems for which a rescheduling solution has been devised, the first solved problem was the *Nurse Scheduling Problem* [25], where the goal is to create a scheduling for nurses working in hospital units: The rescheduling problem [26] deals with the sudden absences of some nurses. Then, the problem of assigning operating rooms to patients, denoted as *Operating Room Scheduling*, has been treated [27]: The rescheduling problem here [28] considers cases in which some patients could not be operated in their assigned slot. More recent problems include the *Chemotherepy Treatment Scheduling* problem [4], in which patients are assigned a chair or a bed for their treatments, the *Rehabilitation Scheduling Problem* [3], which assigns patients to operators in rehabilitation sessions, and the *Master Scheduling Problem* [29], which consists of scheduling different specialties to the operating rooms. The rescheduling solution for the first considers the case of patients unavailability, the second deals with the unavailability of operators and/or the absence of patients [30], while the last [31] considers the unavailability of operating rooms or limitations/changes related to specialties.

## Conclusion

In this paper, we have presented a solution to the Nuclear Medicine Rescheduling problem, as defined in the paper. We first provided a mathematical formulation of the problem, then an encoding based on Answer Set Programming for three scenarios that can motivate the need to reschedule an already computed schedule. Experiments employing real data from a medium size hospital in Italy have shown that our rescheduling solution provides satisfying results even in extreme cases in which the concurrent number of emergencies and unavailability is significant. As future work, we plan to compare our solution to logic-based formalisms other than ASP.

## Data Availability

All the materials to reproduce the experiments can be found at https://github.com/CinziaMarte/JoMS2025.

## Footnotes

1 https://www.salute.gov.it/imgs/C_17_pubblicazioni_3425_allegato.pdf

2 https://ergeagroup.com/it/.

3 https://surgiq.com/.
